# The importance of partnerships in accelerating HIV vaccine research and development

**DOI:** 10.1002/jia2.25824

**Published:** 2021-11-21

**Authors:** Mark B. Feinberg, Nina D. Russell, Robin J. Shattock, Karie B. Youngdahl

**Affiliations:** ^1^ IAVI New York New York USA; ^2^ Bill & Melinda Gates Foundation Seattle Washington USA; ^3^ Imperial College London UK

**Keywords:** AIDS, HIV, partnership, product development, research, vaccine

1

Forty years since the first report of the disease in the United States now known as HIV/AIDS, an efficacious HIV vaccine remains an elusive goal. However, the remarkable level of discovery, innovation and collaboration employed in pursuit of this goal has augmented the scientific insight and capabilities of vaccinology overall and benefitted vaccine development targeting other infectious diseases. Importantly, the progress made in the HIV vaccine research effort is not just a story of the “what” but also the “how.” Given the complexities that make HIV a confoundingly difficult target for vaccine development, and the lack of market opportunity that would encourage pharmaceutical companies to make substantial and sustained at‐risk investments, innovative partnerships for HIV vaccine development have offered a promising path forward. Just as HIV vaccine science has provided the leading edge of vaccine development innovation, the models of collaborative science pioneered in the HIV vaccine field also represent innovations. Indeed, the science and partnership models advancing HIV vaccine development helped to dramatically accelerate the development of highly efficacious SARS‐CoV‐2 vaccines [1, 2]. This viewpoint describes major partnerships and funding models that have driven HIV vaccine discovery and translational science.

Many collaborations have been established over the past 25 years to advance HIV vaccine research by enabling a “big science” approach. Sustained funding and a common strategic focus have enabled greater coordination, faster design and development of immunogens, and more effective sharing of data and technical capabilities.

Chronologically, IAVI (initially known as the International AIDS Vaccine Initiative) was the first of these partnerships to be established. IAVI, founded in 1996, emanated from a call for a new global initiative to accelerate the development of HIV vaccines for worldwide use [3]. IAVI and its major Neutralizing Antibody Center partner, Scripps Research, continue this mandate. HIV vaccine candidates are designed using high‐resolution structure‐based immunogen design techniques with the intent of eliciting broadly neutralizing antibodies (bnAbs). In addition, IAVI, Scripps Research and the Vaccine Research Center at the National Institutes of Health (NIH) are advancing combinations of optimized bnAbs as a potential passive immunization tool with the potential for affordable global access, in partnership with the Serum Institute of India and USAID [4].

The Global HIV Vaccine Enterprise (Enterprise) is an alliance of independent funding, research and advocacy organizations launched in 2005 to mobilize new resources for HIV vaccine research and development (R&D) and promote the acceleration of promising research strategies. The Enterprise, through its first strategic plan, provided a framework that sparked the creation of other major partnerships: in 2005, the Bill & Melinda Gates Foundation (BMGF) launched the Collaboration for AIDS Vaccine Discovery (CAVD) and the NIH‐funded Center for HIV/AIDS Vaccine Immunology (CHAVI).

The CAVD is a network of translational research consortia focused on the design and development of novel vaccine candidates. The CAVD accelerates research through the open sharing of data, and utilization of central service facilities that include best‐in‐class B‐ and T‐cell immunology laboratories, sophisticated mouse models, a data and statistical management centre and a vaccine product development centre that facilitates the translation of promising concepts from the lab into clinical evaluation [5]. This highly collaborative framework has accelerated a range of complex basic, translational and clinical studies that would not have otherwise been possible for individual investigators to pursue.

The CHAVI program founded at Duke University was another multi‐institutional partnership predicated on interdisciplinary and collaborative science. CHAVI investigators have studied virus transmission and elucidated many of the basic aspects of HIV immunology that now inform our mechanistic understanding of requirements for HIV prevention. Building on this effort, in 2012, the NIH funded two Centers for HIV‐AIDS Vaccine Immunology and Immunogen Discovery (CHAVI‐IDs) at Duke University and Scripps Research to focus on HIV immunogen design. In 2019, Scripps and Duke each developed Consortia for HIV/AIDS Vaccine Development (CHAVD), aimed at supporting collaborative research to translate innovative HIV vaccine immunogens into clinical development [6].

The European AIDS Vaccine Initiative (EAVI2020) and the European HIV Vaccine Alliance were established in 2015 with the goal of fostering the development of an effective vaccine. While the European Community previously funded smaller vaccine consortia, these new efforts brought together leading vaccine research groups from academia and industry across Europe. They supported new approaches that bridge laboratory and clinical research studies via iterative vaccine design and optimization programs with the goal of reducing risk of failure in late‐stage development.

ADVANCE (Accelerate the Development of Vaccines and New Technologies to Combat the AIDS Epidemic), a program initiated in 2016 via cooperative agreements between IAVI and the USAID through the United States President's Emergency Plan for AIDS Relief (PEPFAR), has advanced HIV vaccine research and development through partnerships in Africa and India. The foundations of ADVANCE emerged from earlier sustained investments by USAID to establish a network of translational, epidemiological and clinical research centres (CRCs) in five sub‐Saharan African countries and India [7]. Data generated from CRC‐driven epidemiological studies have provided important insights into the process by which bnAbs develop in individuals with HIV — work that is foundational to the design of vaccines aimed at inducing bnAbs for HIV prevention [8, 9].

Table 1 summarizes a variety of partnerships formed to advance HIV prevention research.

**Table 1:** Partnerships

Partnership name	Partners	Year founded	Purpose
IAVI www.iavi.org	IAVI, Rockefeller Foundation (founding donor), BMGF, USAID, others	1996	Translate scientific discoveries into affordable, globally accessible public health solutions
HIV Vaccines Trial Network (HVTN) www.hvtn.org	NIH, Fred Hutchinson Cancer Research Center, others	1999 (The AIDS Vaccine Evaluation Group was funded by NIH in 1988 [11].)	Characterize the safety, immunogenicity and efficacy of HIV vaccine candidates
HIV Prevention Trials Network (HPTN) www.hptn.org	NIH, FHI 360, Fred Hutchinson Cancer Research Center, Johns Hopkins University, Imperial College London	1999 (The HIV Network for Prevention Trials [HIVNET] was funded by DAIDS in 1993 [12].)	Develop and test the safety and efficacy of interventions designed to prevent the acquisition and transmission of HIV
Neutralizing Antibody Consortium	IAVI, Scripps Research, NIH Vaccine Research Center (VRC), others	2002–2008	Discover, describe and optimize HIV bnAbs and develop immunogens to elicit them
Collaboration for AIDS Vaccine Discovery (CAVD) www.cavd.org	BMGF and multiple partners, see [13]	2005	Design a variety of novel HIV vaccine candidates and advance the most promising candidates to clinical trials
IAVI Neutralizing Antibody Center (NAC)	IAVI, Scripps Research, other partners	2008	Discover, describe and optimize HIV bnAbs and develop immunogens to elicit them
CHAVI	NIH, Duke	2005–2012	Undertake the immunologic research required to tackle the major scientific obstacles in the development of an effective HIV vaccine
CHAVI‐ID	NIH, Duke, Scripps Research	2012–2019	Undertake the immunologic research required to tackle the major scientific obstacles in the development of an effective HIV vaccine
IAVI Product Development Center (PDC)	IAVI, BMGF, NIH, others	2013	Support investigators with translation research [14]
European AIDS Vaccine Initiative (EAVI2020) www.eavi2020.org	Imperial College London, University of Oxford, INSERM, others	2015	Advance HIV vaccine concepts that can be moved into clinical trials in a 5‐year window using an experimental medicine research model
ADVANCE www.iavi.org	USAID, IAVI, CRC partners in five African countries and India, others	2016 (with prior, sustained USAID investments starting in 2001)	Support researchers in Africa and India to become leaders in global efforts to identify, evaluate and implement HIV biomedical prevention products
CHAVD www.chavd.org (Scripps) www.dhvi.duke.edu (Duke)	NIH, Duke, Scripps Research	2019	Develop and down select HIV immunogens and regimens that induce bnAb responses for clinical testing
European HIV Vaccine Alliance (EHVA) www.ehv‐a.eu	Horizon 2020, INSERM, Swiss Vaccine Research Institute at the Lausanne University Hospital, others	2016	Develop platforms for HIV vaccine research and engage African scientists to prepare for clinical evaluation of EHVA vaccine candidates
Collaborative HIV Immunogen Project (CHIP)	NIH, BMGF, Scripps CHAVD, Duke CHAVD, HVTN, NIAID Vaccine Research Center, IAVI	2019	Coordinate groups developing HIV vaccine candidates designed to lead to the production of bnAbs [9]

Abbreviations: ADVANCE, Accelerate the Development of Vaccines and New Technologies to Combat the AIDS Epidemic; BMGF, Bill & Melinda Gates Foundation; CHAVI, Center for HIV/AIDS Vaccine Immunology; CHAVI‐ID, Centers for HIV‐AIDS Vaccine Immunology and Immunogen Discovery; CRC, clinical research centre; INSERM, Institut national de la santé et de la recherche médicale; NIAID, National Institute of Allergy and Infectious Diseases; NIH, National Institutes of Health.

Many of the most effective partnerships for HIV prevention research have fostered multidisciplinary science, linking immunology, structural biology, protein science, virology, genetics and expertise in monoclonal antibody discovery and optimization. This collaboration among disciplines is possible only when intra‐ and extra‐institutional barriers are overcome to allow unfettered cooperation, access to scientific core infrastructure and data sharing frameworks to support and connect researchers who, absent these partnerships, are typically more siloed.

Clinical trial networks, such as the HIV Vaccine Trials Network (HVTN) and the HIV Prevention Trials Network, along with other clinical development activities funded by USAID and the European and Developing Countries Clinical Trials Partnership, have enabled the conduct of clinical trials among those most at risk of HIV acquisition. These networks, most specifically HVTN, have developed clinical, laboratory and statistical standards and procedures that advanced the field and, in many cases, allowed the comparison of results from different HIV vaccine studies.

A discussion of partnerships must include mention of their potential complexities and pitfalls. Large collaborative efforts can tend towards groupthink and risk aversion if not actively avoided with diversified funding to generate and support new ideas. Additionally, clinical trial infrastructure established to evaluate late‐stage candidates needs to be repurposed when vaccine candidates do not demonstrate efficacy. Increasingly, the field is focusing on higher through‐put of early‐stage clinical candidates to diversify the pipeline, which will require a selection process that accelerates the most promising concepts. Partnerships should be expanded to include expertise in implementation, delivery and access to HIV vaccines once they are shown to be efficacious. The global disparities in access to SARS‐CoV‐2‐vaccines [10] have highlighted this imperative.

The development of optimal partnerships for the future will require learning from the past. Partnerships formed to advance HIV vaccine research have enabled the global scientific community's rapid pivot to SARS‐CoV‐2 vaccine research [2]. Now, the remarkable display of collaborative science in the response to the COVID‐19 pandemic could “return the favour” to help implementation of more effective partnerships to expedite HIV vaccine development. With study of how COVID‐19 partnerships have succeeded, especially in linking pharmaceutical companies with the public and academic sectors, we can ensure that our partnerships are robust and resilient enough to solve the challenges that remain on the path to an HIV vaccine.

## COMPETING INTERESTS

MBF and KBY are affiliated with IAVI. NDR is affiliated with the Bill & MG Foundation. RJS is affiliated with the European AIDS Vaccine Initiative.

## AUTHORS’ CONTRIBUTIONS

MBF, NDR, RJS and KBY contributed to the conceptualization and writing of this manuscript.

## FUNDING

None.

## References

[1] Fauci AS . The story behind COVID‐19 vaccines. Science. 2021;372(6538):109. 33833099 10.1126/science.abi8397

[2] McMahon JH , Hoy JF , Kamarulzaman A , Bekker LG , Beyrer C , Lewin SR . Leveraging the advances in HIV for COVID‐19. Lancet North Am Ed. 2020;396(10256):943–4. 10.1016/S0140-6736(20)32012-2 PMC7529395 33010825

[3] HIV Vaccines . Accelerating the development of preventive HIV vaccines for the world. Rockefeller Foundation; 1994.

[4] IAVI and Scripps Research join efforts with NIH to expedite development of globally accessible and affordable HIV antibody combination products [Internet]. IAVI. [cited 2021 June 11] Available from: https://www.iavi.org/news‐resources/press‐releases/2020/iavi‐scripps‐nih‐partnerships‐hiv‐antibodies

[5] Collaboration for AIDS Vaccine Discovery . About the grantees. [Internet]. CAVD. [cited 2021 May 30] Available from:https://www.cavd.org/grantees/Pages/default.aspx

[6] Duke Human Vaccine Institute . Duke Consortia for HIV/AIDS Vaccine Development. [Internet]. Duke University School of Medicine. [cited 2021 June 11]. Available from: https://dhvi.duke.edu/our‐programs/duke‐consortia‐hivaids‐vaccine‐development‐chavd

[7] IAVI Clinical and Epidemiology Research . Advance [Internet]. IAVI. [cited 2021 May 30]. Available from: https://www.iavi.org/our‐work/clinical‐epidemiology‐research/advance

[8] Price MA , Kilembe W , Ruzagira E , Karita E , Inambao M , Sanders EJ , et al. Cohort profile: IAVI's HIV epidemiology and early infection cohort studies in Africa to support vaccine discovery. Int J Epidemiol. 2021; 50(1):29–30. 32879950 10.1093/ije/dyaa100 PMC7938500

[9] Haynes BF , Burton DR , Mascola JR . Multiple roles for HIV broadly neutralizing antibodies. Sci Transl Med. 2019; 11(516). 10.1126/scitranslmed.aaz2686 PMC7171597 31666399

[10] Rouw A , Wexler A , Kates J , Michaud J . Global COVID‐19 vaccine access: a snapshot of inequality [Internet]. Kaiser Family Foundation portal. [cited 2021 May 30] Available from: https://www.kff.org/policy‐watch/global‐covid‐19‐vaccine‐access‐snapshot‐of‐inequality

[11] National Institute of Allergy and Infectious Diseases History of HIV vaccine research [Internet]. US National Institutes of Health; 2018. [cited 2021 July 4]. Available from: https://www.niaid.nih.gov/diseases‐conditions/hiv‐vaccine‐research‐history

[12] Office for the Assistant Secretary of Planning and Evaluation . An inventory of federally sponsored HIV and HIV‐relevant databases. Database: HIV Vaccine Clinical Trials Network (HVTN) and HIV Prevention Clinical Trials Network (HPTN) ‐ Formerly HIV Network for Prevention Trials (HIVNET) [Internet]. US Department of Health and Human Services. [cited 2021 July 4]. Available from: https://aspe.hhs.gov/report/inventory-federally‐sponsored‐hiv‐and‐hiv‐relevant‐databases/database‐hiv‐vaccine‐clinical‐trials‐network‐hvtn‐and‐hiv‐prevention‐clinical‐trials‐network‐hptn‐formerly‐hiv‐network‐prevention

[13] The central service facilities for CAVD include: IAVI (VxPDC), Fred Hutchinson Cancer Research Center, Vaccine Immunology Statistical Center (VISC), Scripps Electron Microscopy Characterization Center (EMC2), Duke University Comprehensive Antibody Vaccine Immune Monitoring Consortium (CA‐VIMC), NIH Vaccine Research Center Comprehensive Cellular Vaccine Immune Monitoring Consortium (CCVIMC) . A link to the description of all other CAVD partners can be found online. [cited 2021 October 19]. Available from:https://www.cavd.org/about/Pages/CollaboratingInstitutions_alpha.aspx

[14] IAVI's Product Development Center . Translation & product development [Internet]. IAVI. [cited 2021 May 30]. Available from: https://www.iavi.org/our‐work/translation‐product‐development

